# Frequency Tuning of Graphene Nanoelectromechanical Resonators via Electrostatic Gating

**DOI:** 10.3390/mi9060312

**Published:** 2018-06-20

**Authors:** Tengda Mei, Jaesung Lee, Yuehang Xu, Philip X.-L. Feng

**Affiliations:** 1 School of Electronic Science and Engineering, University of Electronic Science and Technology of China, Chengdu 611731, China; txm343@case.edu; 2 Electrical Engineering, Case School of Engineering, Case Western Reserve University, Cleveland, OH 44106, USA; jaesung.lee@case.edu

**Keywords:** nanoelectromechanical systems (NEMS), graphene resonators, electrostatic gate tuning, frequency tuning model

## Abstract

In this article, we report on a comprehensive modeling study of frequency tuning of graphene resonant nanoelectromechanical systems (NEMS) via electrostatic coupling forces induced by controlling the voltage of a capacitive gate. The model applies to both doubly clamped graphene membranes and circumference-clamped circular drumhead device structures. Frequency tuning of these devices can be predicted by considering both capacitive softening and elastic stiffening. It is shown that the built-in strain in the device strongly dictates the frequency tuning behavior and tuning range. We also find that doubly clamped graphene resonators can have a wider frequency tuning range, while circular drumhead devices have higher initial resonance frequency with same device characteristic parameters. Further, the parametric study in this work clearly shows that a smaller built-in strain, smaller depth of air gap or cavity, and larger device size or characteristic length (e.g., length for doubly clamped devices, and diameter for circular drumheads) help achieve a wider range of electrostatic frequency tunability. This study builds a solid foundation that can offer important device fabrication and design guidelines for achieving radio frequency components (e.g., voltage controlled oscillators and filters) with the desired frequencies and tuning ranges.

## 1. Introduction

Two-dimensional (2D) materials such as graphene and atomic layer semiconductors have recently attracted tremendous attention and research interest due to their unconventional and extraordinary material properties stemming from their atomically thin layered structures. In addition to excellent and unique properties in optical [1] and electrical domains (e.g., mobility of 1,000,000 cm^2^/(V⋅s) [2]), graphene also exhibits remarkable mechanical properties such as ultrahigh Young’s modulus *E*_Y_ = 1 TPa, and a large breaking strain limit up to 25% [3,4]. These attributes have made graphene an attractive and promising candidate for highly miniaturized and aggressively scaled resonant-mode nanoelectromechanical systems (NEMS) with unprecedented device performance. To date, mainly doubly clamped [5,6,7,8] membranes and ribbons, and circumference-clamped circular drumhead graphene resonators [9,10,11,12,13,14,15,16] have been prototyped, and fundamental device physics and basic device characteristics have been studied. Further, potential applications of these graphene 2D NEMS resonators in sensing of external stimuli and perturbations [17], and components for radio frequency (RF) signal processing and communications (e.g., oscillators [11]), have been attempted. For these applications, continuous and wide frequency tuning controlled by moderate level electrical signals (i.e., voltage or current) is highly desirable, to render these systems tunable, flexible, or even programmable and reconfigurable. Indeed, strong frequency tunability has been quite heavily pursued in more conventional and state-of-the-art microelectromechanical systems (MEMS) based resonators and oscillators; however, tuning range is often limited up to 5% due to their high stiffness [18]. Thanks to ultra-strong yet highly stretchable crystals and their related material properties, graphene NEMS resonators can exhibit remarkably broad tunability of resonance frequency, with Δ*f*_res_/*f*_res_ > 300% [12]. 

Among various device platforms and resonance excitation and detection methods, including photothermal and electrothermal schemes, electrostatic excitation and control of graphene 2D NEMS devices are always attractive for on-chip integration with mainstream technologies, and are particularly promising toward achieving wide frequency tuning ranges [6,7,8,9,10,11,12]. In the electrostatic scheme, device structures form a capacitor between a suspended 2D material and a bottom (or top) gate, enabling electrostatic control of electrical and mechanical device performance by applying direct current (DC) electric potential (i.e., DC gate voltage). The tunable conductance with respect to the applied DC potential at the gate offers strong coupling between the mechanical motion and the electrical conductance, enabling mechanical motion detection via sensitive vibrating channel field effect transistor (VCFET) signal transduction [6]. Further, the electrostatic scheme only requires ultralow power consumption owing to lack of DC current flowing between the suspended graphene NEMS and the gate. More importantly, in the electromechanical domain, frequency tuning can be easily achieved by applying the gate DC voltage, which modifies the effective spring constants of the devices by electrostatic-mechanical coupling. The reported frequency tuning results in the electrostatic scheme to date, however, exhibit complicated behavior including frequency downshifts and upshifts [6,7,8,9,10,11,12,13,14,16]. Several attempts have been made to develop frequency tuning models that can be used to describe and explain the precise tuning mechanisms of devices [6,9,10,19,20,21,22,23,24]. So far, however, these models are not sufficient to comprehensively explain the complicated frequency tuning behavior and the device coupling mechanisms. Thus, it can be quite challenging to understand contributions from various parameters, including device geometry and built-in tension (strain) on the frequency tuning ranges. 

In this work, we present an accurate frequency tuning model for electrostatically actuated doubly clamped membranes and circumference-clamped circular drumhead graphene resonators. This model can quantitatively clarify various mechanisms of frequency tuning. We carefully develop the frequency tuning model via electrostatic gating voltage by considering multiphysics coupling of elastic and electrostatic effects. Our analysis reveals that various device parameters strongly govern the device resonance and its frequency tuning range. Moreover, we find that frequency tuning range and wide-range tuning behavior can be finely engineered by controlling important parameters such as the built-in strain. 

## 2. Analytical Model and Computational Methods

### 2.1. Development of Frequency Tuning Model and Analysis Procedure

**Figure 1:**
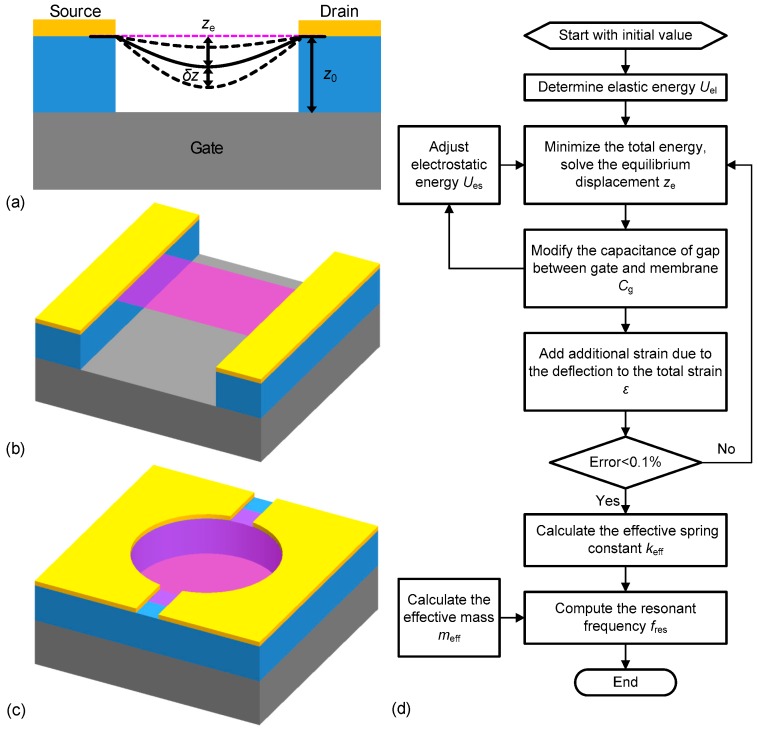
Device structures and modeling procedure of electrostatic tuning of resonance frequency in graphene nanoelectromechanical systems (NEMS). (**a**) Deflection of graphene resonator under electrostatic force. Without gate voltage, the membrane is flat (thin magenta dashed line) and suspended over the trench at equilibrium. With applied DC gate voltage, the membrane deflects to new equilibrium displacement *z*_e_ (thick black solid line). With AC actuation, the membrane vibrates with amplitude *δz* (thick black dashed lines). (**b**,**c**) Schematics of doubly clamped and circular drumhead graphene resonators, respectively. (**d**) Flowchart for computational implementation of the frequency tuning model.

We start to develop our model by calculating the deflection of the suspended graphene as a thin membrane subject to external forces. We assume that the suspended 2D graphene membrane has negligible flexural rigidity, and it can be stretchable by transversely pulling the membrane using the electrostatic force induced by a gate voltage. Figure 1a shows the side-view schematic of the deflection of the graphene resonator, and Figure 1b,c illustrate the schematics of a doubly clamped membrane and the circumference-clamped circular drumhead device structure, respectively. The deflection of the 2D material is precisely computed by following the procedure outlined in the flowchart shown in Figure 1d. The equilibrium displacement (*z*_e_) on the center of the devices is solved by minimizing the total energy (sum of elastic energy *U*_el_ and electrostatic energy *U*_es_) upon the application of electrostatic force. To make the model accurate, we perform several iterative calculations of the capacitance between the gate and suspended membrane *C*_g_, equilibrium displacement *z*_e_, and total strain *ε* after deflection. Substituting the modified strain *ε* and *C*_g_ into the effective spring constant *k*_eff_, the resonance frequency *f*_res_ can then be obtained using a relation of *f*_res_ = (*k*_eff_/*m*_eff_)^1/2^/2*π*, with the calculated effective mass of resonance mode, *m*_eff_. 

### 2.2. Frequency Tuning of Doubly Clamped Graphene Resonator

Frequency tuning in a doubly clamped graphene resonator under electrostatic coupling can be calculated by examining the elastic energy of the suspended graphene and electrostatic energy in the device capacitor. Consider the doubly clamped device has a static deflection profile of *u*_d_(*x*) = 4*z*_e_(*Lx* − *x*^2^)/*L*^2^, (0 < *x* < *L*), where *L* is the length of the suspended graphene membrane (or ribbon), and it has the maximum static deflection of *z*_e_ at the midpoint (*x* = *L*/2) due to the center of the symmetric structure. Under uniformly distributed force, we neglect the Poisson’s ratio in doubly clamped case since the resonance frequency is independent on width of the membrane. For the doubly clamped device structure shown in Figure 1b, the elastic energy stored in the stretched membrane *U*_el,d_ can be described as [24]

(1) $$U_{\mathrm{el},d}=\int_{0}^{w}\int_{0}^{L}\frac{\gamma }{2}u\prime^{2}\left(x\right)dxdy=\int_{0}^{w}\int_{0}^{L}u\prime^{2}\left(x\right)\left[\frac{E_{Y}t\varepsilon_{0}}{2}\text{​}+\frac{E_{Y}t}{4L}\int_{0}^{L}u\prime^{2}\left(\chi \right)d\chi \right]dxdy$$

where *w*, *E*_Y_, *t*, and *ε*_0_ are the width, Young’s modulus, thickness, and built-in strain of membrane, respectively. The total tension after deflection is *γ* = *E*_Y_*tε*_d_*,* where *ε*_d_ is the total strain that is composed of both the built-in strain and the added strain according to the deflection. By introducing deflection profile *u*_d_(*x*) into Equation (1), the elastic energy is then

(2) $$U_{\mathrm{el},d}=\frac{64E_{Y}twz_{e}{}^{4}}{9L^{3}}+\frac{8E_{Y}tw\varepsilon_{0}z_{e}{}^{2}}{3L}.$$

We consider the suspended graphene as a capacitor where the graphene is a deformable electrode, the bottom back gate is a fixed plate electrode, and the air gap is the dielectric layer. Considering deflection profile, the device capacitance *C*_g,d_ is

(3) $$C_{g,d}=\int_{0}^{w}\int_{0}^{L}\frac{{є}_{0}}{z_{0}-z_{e}\frac{4}{L^{2}}(Lx-x^{2})}dxdy,$$

where ${є}_{0}$ is the permittivity, and *z*_0_ is the depth of the air gap. The electrostatic energy stored in the device structure is then

(4) $$U_{\mathrm{es}}=-\frac{1}{2}C_{g,d}V_{g}^{2},$$

where *V*_g_ is the gate voltage. The equilibrium displacement *z*_e_ can be obtained by minimizing the total energy thus finding the deflection position where the elastic and electrostatic forces are equal:

(5) $$\frac{\partial (U_{\mathrm{el},d}+U_{\mathrm{es}})}{\partial z_{e}}=\frac{256E_{Y}twz_{e}{}^{3}}{9L^{3}}+\frac{16E_{Y}wt\varepsilon_{0}z_{e}}{3L}-\frac{1}{2}C_{g}^{\prime }V_{g}{}^{2}=0.$$

Although Equation (3) can be further simplified by expanding it to *C*_g,d_ = ${є}_{0}$*wL*/*z*_0_ + 2${є}_{0}$*wLz*_e_/3$z_{0}^{2}$ + 8*є*_0_*wLz*_e_^2^/15$z_{0}^{3}$ + …, and the initial three terms of the series can be used for calculating *z*_e_ using Equation (5), this calculation produces an approximated value of *z*_e_ and it may give quite large error, especially when we calculate the exponent of *z*_e_. Instead, here we perform several iterations to find accurate device *C*_g_ by repeating a process of plugging *z*_e_ obtained using Equation (5) back into Equation (3) till it is convergent. The total strain *ε*_d_ in the graphene includes built-in strain and the added strain upon deformation. The added strain after deflection is obtained by comparing the extended length from the deformed graphene sheet and the initial graphene length. The total strain is

(6) $$\varepsilon_{d}=\frac{1+\varepsilon_{0}}{L}\;\int_{0}^{L}\sqrt{1+{\left(\frac{\partial u}{\partial x}\right)}^{2}\;}dx-\;1=\;\frac{1+\varepsilon_{0}}{2}\sqrt{1+\frac{16z_{e}{}^{2}}{L^{2}}}+\frac{(1+\varepsilon_{0})L}{8z_{e}}\ln(\sqrt{1+\frac{16z_{e}{}^{2}}{L^{2}}\;}\;+\;\frac{4z_{e}}{L})-1.$$

Next, we consider the dynamic motion of the doubly clamped graphene membrane vibrating at its resonance frequency. The mode shape of the fundamental mode resonance of doubly clamped devices can be expressed by a sinusoidal function. Considering the clamped boundary conditions, we obtain the mode shape of fundamental resonance *u*_f,d_(*x*) = *δz*sin(*πx*/*L*), where *δz* is the dynamic displacement at the midpoint. We assume that at the small static defection *z*_e_ of the graphene membrane, the vibration mode shape remains to be in the sinusoidal form. The elastic energy *δU*_el_ for the fundamental mode resonance of the strained graphene resonator is

(7) $$\delta U_{\mathrm{el},d}=\int_{0}^{w}\int_{0}^{L}\frac{\gamma }{2}u_{f,d}^{\prime }{}^{2}(x)dxdy=\frac{\pi^{2}E_{Y}tw\varepsilon_{d}}{4L}{\left(\delta z\right)}^{2}.$$

The effective spring constant *k*_eff,d_ can be given by the second order differentiation of the total energy:

(8) $$k_{\mathrm{eff},d}=\frac{\partial^{2}\delta U_{\mathrm{el},d}}{\partial \delta z^{2}}+\frac{\partial^{2}U_{\mathrm{es}}}{\partial z_{e}^{2}}=\frac{\pi^{2}E_{Y}wt\varepsilon_{d}}{2L}-\frac{8{є}_{0}wL}{15z_{0}^{3}}V_{g}^{2}.$$

Resonance frequency is determined by *f*_res_ = (1/2*π*)(*k*_eff_/*m*_eff_)^1/2^, where *m*_eff_ is effective mass which can be calculated from kinetic energy of membrane at resonance. The peak kinetic energy *E*_kin,d_ is

(9) $$E_{\mathrm{kin},d}=\frac{1}{2}m_{\mathrm{eff},d}{\left(\delta z\right)}^{2}=\frac{1}{2}\rho t\int_{0}^{w}\int_{0}^{L}{\left(\delta z\sin\left(\frac{\pi }{L}x\right)\right)}^{2}dxdy=\frac{1}{4}\rho twL{\left(\delta z\right)}^{2}.$$

From Equation (9), the effective mass of the fundamental mode is *m*_eff,d_ = 0.5*ρtwL* in doubly clamped case, where *ρ* is the mass density. The frequency tuning for the doubly clamped resonator can be given by

(10) $$f_{\mathrm{res},d}=\frac{1}{2\pi }\sqrt{\frac{k_{\mathrm{eff},d}}{m_{\mathrm{eff},d}}}=\frac{1}{2\pi }\sqrt{\frac{\pi^{2}E_{Y}\varepsilon_{d}}{\rho L^{2}}-\frac{16{є}_{0}}{15\rho tz_{0}^{3}}V_{g}^{2}}.$$

### 2.3. Circumference-Clamped Circular Membrane

We now turn to analyze the frequency tuning behavior of the circular drumhead graphene resonator. For convenience, we use a polar coordinate for the circular drumhead that closely matches the device geometry. The elastic energy stored in the stretched membrane *U*_el,c_ can be obtained by [19,25]

(11) $$U_{\mathrm{el},c}=\int_{0}^{2\pi }\int_{0}^{R}\left\{\frac{E_{Y}t}{2}[\varepsilon_{0,r}{\left(\frac{\partial u}{\partial r}\right)}^{2}+\frac{\varepsilon_{0,\theta }}{r^{2}}{\left(\frac{\partial u}{\partial \theta }\right)}^{2}]\;+\;\frac{E_{Y}t}{8(1-v^{2})}{\left(\frac{\partial^{2}u}{\partial r^{2}}\right)}^{2}\right\}rdrd\theta ,$$

where *R*, *ν*, *ε*_0,*r*_, *ε*_0,*θ*_ are radius, Poisson’s ratio, initial radial strain, initial tangential strain, respectively. Similar to the doubly clamped case, we assume that the curvature of the static deflection forms the parabolic shape, and it has the maximum static deflection at its center due to symmetry. The solution of deflection can also be *u*_c_(*r*) = *z*_e_(*R*^2^ − *r*^2^)/*R*^2^, where *z*_e_ is the static defection at the center of the drumhead. The elastic energy induced by electrostatic deformation is

(12) $$U_{\mathrm{el},c}=\frac{2\pi E_{Y}tz_{e}^{4}}{3(1-v^{2})R^{2}}+\pi E_{Y}t\varepsilon_{0,r}z_{e}^{2}+\frac{\pi E_{Y}t\varepsilon_{0,r}R^{2}}{2(1-v^{2})}.$$

The device capacitance between the suspended graphene and the bottom gate is

(13) $$C_{g,c}=\;\int_{0}^{2\pi }\int_{0}^{R}\frac{{є}_{0}}{z_{0}-z_{e}\frac{1}{R^{2}}(R^{2}-\;r^{2})}rdrd\theta ,$$

and the equilibrium displacement *z*_e_ can be obtained by minimizing the total energy

(14) $$\frac{\partial (U_{\mathrm{el},c}+U_{\mathrm{es}})}{\partial z_{e}}=\frac{8\pi E_{Y}tz_{e}^{3}}{3(1-v^{2})R^{2}}+2\pi E_{Y}t\varepsilon_{0,r}z_{e}-\frac{1}{2}C_{g}^{\prime }V_{g}^{2}=0.$$

To find accurate *z*_e_, we conduct iterations using Equations (12)–(14) till it is convergent. Based on the deflection curvature and estimated *z*_e_, the total radial strain from stretching of the drumhead is estimated by calculating the radial elongation of the membrane:

(15) $$\varepsilon_{r}=\frac{1+\varepsilon_{0,r}}{2R}\int_{-R}^{R}\sqrt{1+{\left(\frac{\partial u}{\partial r}\right)}^{2}}dr-\;1=\frac{1+\varepsilon_{0,r}}{2}\sqrt{1+\frac{4z_{e}^{2}}{R^{2}}}+\frac{(1\;+\;\varepsilon_{0,r})R}{4z_{e}^{2}}\ln(\sqrt{1\;+\;\frac{4z_{e}^{2}}{R^{2}}}\;+\;\frac{2z_{e}}{R})\;-\;1\;.$$

The fundamental mode resonance and its mode shape of circular membrane can be expressed using the 0th-order Bessel function of the first kind, *J*_0_. Considering the circumference-clamped boundary conditions, the mode shape of the fundamental resonance is *u_f_*_,c_(*r*) = *δzJ*_0_(2.405*r*/*R*). The elastic energy of the strained circular drumhead at resonance is then modified by substituting the mode shape *u_f_*_,c_(*r*) into Equation (11):

(16) $$\delta U_{\mathrm{el},c}=0.271\frac{2.405^{2}\pi E_{Y}t\varepsilon_{r}}{2}{\left(\delta z\right)}^{2}+\frac{\pi E_{Y}t\varepsilon_{r}R^{2}}{2(1-v^{2})}.$$

With capacitive softening, the effective spring constant for the circular drumhead is given by

(17) $$k_{\mathrm{eff},c}=\;\frac{\partial^{2}\delta U_{\mathrm{el},c}}{\partial \delta z^{2}}\;+\;\frac{\partial^{2}U_{\mathrm{es}}}{\partial z_{e}^{2}}\;=\;4.924E_{Y}t\varepsilon_{r}\;-\;\frac{{є}_{0}\pi R^{2}}{3z_{0}^{3}}V_{g}^{2}.$$

The effective mass of the device can be calculated from the peak kinetic energy,

(18) $$E_{\mathrm{kin},c}=\frac{1}{2}m_{\mathrm{eff},c}{\left(\delta z\right)}^{2}=\frac{1}{2}\rho t\int_{0}^{2\pi }\int_{0}^{R}{\left(\delta zJ_{0}\left(\frac{2.4}{R}r\right)\right)}^{2}rdrd\theta =0.136\pi \rho tR^{2}{\left(\delta z\right)}^{2}.$$

We obtain the effective mass *m*_eff_ = 0.271*ρtπR*^2^ for the fundamental mode resonance in the circular drumhead case. Thus, the resonance frequency for the circular drumhead under electrostatic gating is given by

(19) $$f_{\mathrm{res},c}=\;\frac{1}{2\pi }\sqrt{\frac{k_{\mathrm{eff},c}}{m_{\mathrm{eff},c}}}\;=\;\frac{1}{2\pi }\sqrt{\frac{2.4^{2}E_{Y}\varepsilon_{r}}{\rho R^{2}}\;-\;\frac{{є}_{0}}{0.813\rho tz_{0}^{3}}V_{g}^{2}}.$$

## 3. Results and Discussions

**Figure 2:**
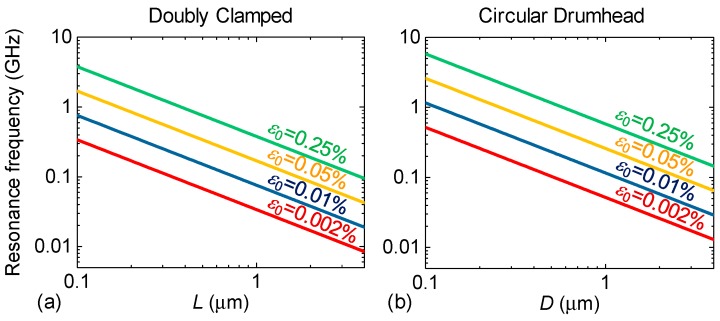
Calculated resonance frequencies of single-layer (1L) graphene resonators without applying gate voltage. Due to photoresist residue in fabrication and surface absorption, we assume the mass is higher than the intrinsic device mass (of carbon atoms only), and we take an effective mass ratio of 2 as the typical value. Frequency scaling of (**a**) doubly clamped membranes and (**b**) circular drumhead graphene resonators. The labels represent built-in strain levels in the devices.

We first calculate frequency scaling of the single layer (1L) graphene resonators without the external gate voltage to understand effects of device dimension and built-in strain on the resonance frequency. Figure 2 shows the simulated frequency scaling of doubly clamped and circular drumhead graphene resonators using Equations (10) and (19) at zero gate voltage *V*_g_ = 0 V as a function of device characteristic dimension (length for the doubly clamped structures, and diameter for the circular drumhead devices) with different built-in strain levels of 0.002%, 0.01%, 0.05%, and 0.25%, respectively. Due to possible photoresist residue on the device during fabrication and surface adsorbates, we assume the mass is higher than the intrinsic device mass estimated by device dimensions and mass density, and we take an effective mass ratio of 2 (i.e., *m*_device_ = 2 × *m*_graphene_) as a typical value. With strain levels from 0.002% to 0.25%, the resonance frequency depends on the length for the doubly clamped case and the diameter for the circular drumhead case, with *f*_res_~*L*^−1^, and *f*_res_~*D*^−1^ power laws, at zero bias condition. It is clearly shown that in both doubly clamped and circular drumhead cases, resonance frequency increases as the characteristic device dimension decreases and as built-in strain increases. For the same characteristic length and built-in strain, the circular device gives higher resonance frequency than the doubly clamped device does, since its circumference-clamped structure provides higher spring constant and smaller effective mass. These correlations for both cases agree with the results from previous models [6,9,10,19,20,21,22,23,24] at gate voltage *V*_g_ = 0 V. To enable resonance frequency above gigahertz, *f*_res_ > 1 GHz, built-in strain ≈0.25% with length smaller than 0.4 μm or built-in strain ≈0.05% with length smaller than 0.18 μm are required for doubly clamped graphene resonators. For circular drumhead resonators to achieve *f*_res_ > 1 GHz, it needs a built-in strain ≈0.05% with a diameter smaller than 0.22 μm or a built-in strain ≈0.25% with a diameter smaller than 0.6 μm. Accordingly, fabricating smaller size membranes, larger built-in strain, and circular drumhead structure are preferred to attain higher resonance frequency. 

**Figure 3:**
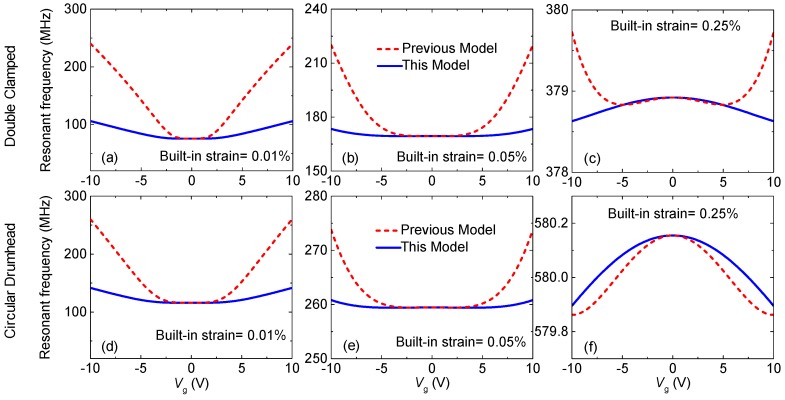
Three typical frequency tuning behaviors for both a doubly clamped graphene resonator (upper row) and a circular drumhead graphene resonator (lower row) with varying built-in strain of (**a**,**d**) 0.01%, (**b**,**e**) 0.05%, and (**c**,**f**) 0.25%. The red dashed lines show previous modeling and blue solid lines show this model by assuming that effective mass ratio = 2, air gap *z*_0_ = 300 nm, length *L* = 1 μm and diameter *D* = 1 μm, respectively.

Now we focus on electrostatic frequency tuning with respect to gate voltage *V*_g_. At different built-in strain levels, we find three frequency tuning behaviors: resonance frequency increases monotonically with |*V*_g_|, it decreases monotonically with |*V*_g_|, and it first decreases, then increases with increasing |*V*_g_|. All these cases are observed in existing experiments [5,6,7,8,9,10,11,12,13,14,15,16] for both of doubly clamped and circular drumhead graphene resonators. Figure 3 shows simulated frequency tuning of the doubly clamped and circular drumhead single-layer (1L) graphene resonators, with typical effective mass ratio = 2, air gap *z*_0_ = 300 nm, length *L* = 1 μm and diameter *D* = 1 μm, respectively. The results show that a big difference between our model and the previous modeling. For the device with relatively small built-in strain (*ε*_0_ = 0.01% in Figure 3a,d), applying |*V*_g_| leading to elastic stiffening which is much bigger than that of capacitive softening, causing frequency elevation (“U” shape). Whereas for the device with large built-in strain (*ε*_0_ = 0.25% in Figure 3c,f), the capacitive softening dominates as |*V*_g_| increases, which keeps reducing the resonance frequency. For intermediate built-in strain (*ε*_0_ = 0.05% in Figure 3b,e), initially the capacitive softening dominates, then the spring constant stiffening dominates as |*V*_g_| increases, showing “W” shape frequency tuning. These tuning behaviors also affect frequency tuning range of the graphene resonators. For smaller built-in strain (*ε*_0_ = 0.01%), the resonance frequency of the doubly clamped device (*L* = 1 μm, *z*_0_ = 300 nm) shifts from 75 MHz to 98 MHz when |*V*_g_|= 0 to 10 V (Figure 3a), showing the frequency tuning range of Δ*f*_res_/*f*_res_ ≈ 31%. For the device with the higher built-in strain (*ε*_0_ = 0.25%), although it leads to higher initial resonance frequency, the frequency shift is very small (378.9 MHz to 378.3 MHz), offering very limited frequency tunability, Δ*f*_res_/*f*_res_ ≈ 0.15%. These results suggest that there is an important trade-off between achieving high initial resonance frequency and wide frequency tuning range, which should be considered for designing the desired device performance for the specific applications. For example, devices with large initial strain are preferred for high frequency signal processing applications, and small initial strain facilitates realizing voltage controlled tunable devices that could be useful for making systems with tunable or programmable functions. 

**Figure 4:**
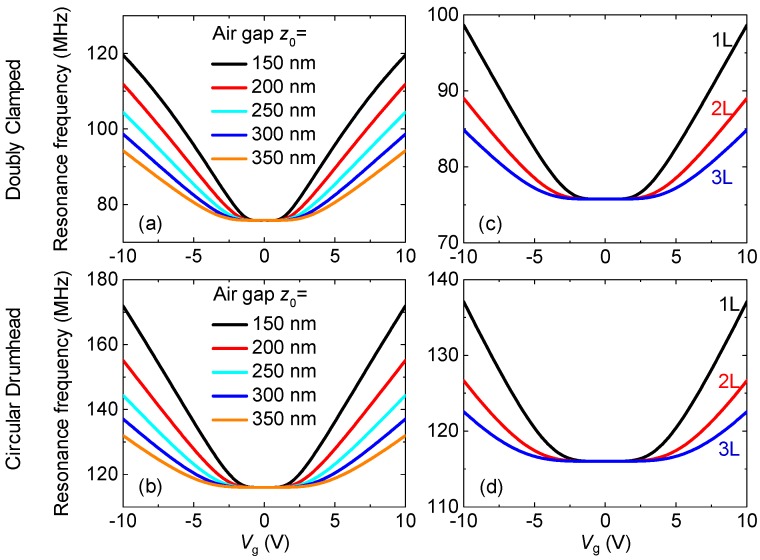
Dependence of resonance characteristics on depth of air gap and number of layers. Frequency tuning of doubly clamped graphene resonators with varying (**a**) air gap and (**c**) number of layers. Simulated resonance frequencies and tuning characteristics for circular drumhead resonators with (**b**) different depth of air gap and (**d**) number of layers.

We also study frequency tuning with varying device parameters such as the depth of the air gap and the number of layers in the doubly clamped and circular drumhead resonators. Figure 4a,b shows that the device (*L* = 1 μm, *ε*_0_ = 0.01%) with the smaller air gap can provide much larger frequency tuning range for the doubly clamped and circular drumhead resonators (e.g., Δ*f*_res_/*f*_res_ ≈ 24.4% for *z*_0_ = 350 nm, and Δ*f*_res_/*f*_res_ ≈ 57.8% for *z*_0_ = 150 nm, for doubly clamped devices). Interestingly, with the same device parameters, such as the characteristic length, initial strain, depth of air gap, doubly clamped devices show larger tuning ranges when the gate voltage is changed (|*V*_g_| = 0–10 V) (Δ*f*_res_/*f*_res_ ≈ 57.8% for *L* = 1 μm, *z*_0_ = 150 nm, *ε*_0_ = 0.01%), compared to those of the circular drumhead resonators (Δ*f*_res_/*f*_res_ ≈ 48.2% for *D* = 2*R* = 1 μm, *z*_0_ = 150 nm, *ε*_0_ = 0.01%). This is attributed to the fact that the fully clamped circular drumhead devices have larger stiffness at the beginning, making it less responsive to incremental external electrostatic force.

Further, we have investigated the dependence of resonance characteristics on a number of layers. Figure 4c,d shows frequency tuning in single-layer (1L) and few-layer graphene doubly clamped and circular drumhead resonators. In the same device parameters (*L* = 1 μm, *ε*_0_ = 0.01%, *z*_0_ = 300 nm) without an applied gate voltage, the resonance frequencies are independent of the number of layers. With the gate voltage, the resonance frequency for the 1L graphene resonators exhibits a much higher frequency tuning capability, compared to those in the few-layer graphene resonators (Δ*f*_res_/*f*_res_ ≈ 30.2% for 1L, Δ*f*_res_/*f*_res_ ≈ 17.5% for 2L, and Δ*f*_res_/*f*_res_ ≈ 12.0% for 3L doubly clamped graphene resonators), and we also find a similar trend in the circular drumhead resonators. According to Equations (9) and (19), the elastic stiffening is independent of the thickness *t*, while the capacitance softening increases with the increasing thickness of the membrane. For the built-in strain ≈0.01% in these cases, the softening effects of the few-layer are stronger compared to the softening in the single layer. Therefore, 1L graphene resonators give a much higher frequency at the same voltage and thus a wider tuning capability, compared to those in the few-layer graphene resonators.

**Figure 5:**
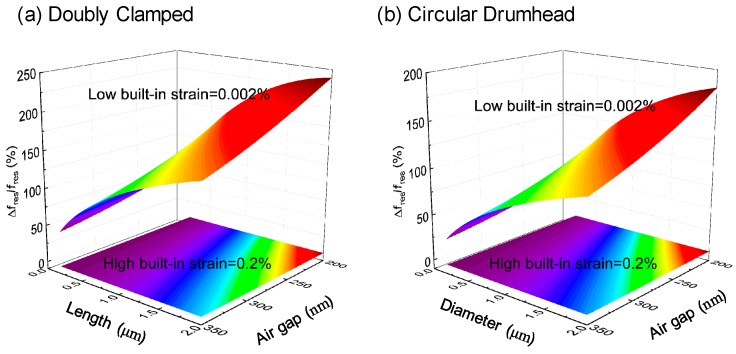
3D plots of computed frequency tuning with varying characteristic dimension and air gap with high built-in strain (0.2%) and low built-in strain (0.002%). (**a**) Frequency tunability of doubly clamped single-layer (1L) graphene resonators, and (**b**) circular drumhead 1L graphene resonators.

We plot the frequency tuning range to clearly display tuning capability with respect to important parameters, including characteristic dimension, depth of air gap, and strain (Figure 5). Figure 5 shows the frequency tuning at gate voltage from *V*_g_ = 0 V to 10V as a function of the characteristic dimension and air gap with high built-in strain (0.2%) and low built-in strain (0.002%), for both doubly clamped and circular drumhead graphene resonators, respectively. With an air gap from 200 nm to 350 nm, length or diameter from 0.2 μm to 2 μm, and lower built-in strain (0.002%), this gives a larger frequency tuning range, while a higher built-in strain (0.2%) leads to a smaller frequency tuning range for both types of geometries for the graphene resonators. We find that a smaller depth of air gap, smaller initial strain, and longer characteristic dimension help achieve a wide frequency tuning range.

**Figure 6:**
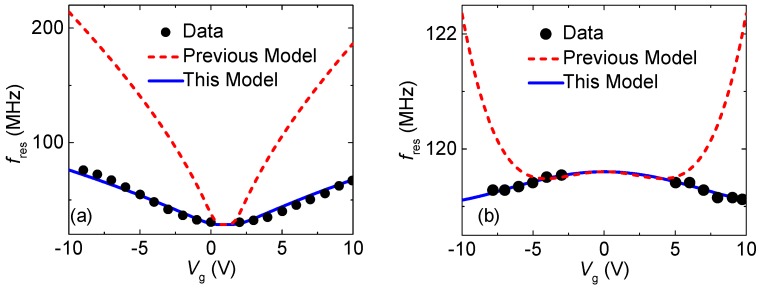
Fitting frequency tuning model to experimental data. The measured data from [6] and fitted data with our modeling (blue solid lines) and previous modeling (red dashed lines) [24] by assuming that built-in strain *ε*_0_, effective mass ratio, length *L*, air gap *z*_0_ are (**a**) 0.003%, 3.5, 1.1 μm 250 nm and (**b**) 0.23%, 5.7, 1.8 μm 250 nm, respectively.

Figure 6 shows the experimental frequency tuning data from [6] and fitted results using our model (blue solid lines) and previous modeling (red dashed lines) [24]. Due to the trapped charges in the membrane, the symmetry axis of the frequency tuning curve is shifted from zero gate voltage. The results show that our model agrees well with experimental data and it is much more accurate compared to that of the previous modeling, particularly at a high voltage regime. In a previous model [24], the equilibrium displacement *z*_e_, which is a positive correlate to the strain, depends on the DC gate voltage *V*_g_, with *z*_e_~$V_{g}^{2}$ power laws [6,10]. This is the only correct form for sufficiently small *V*_g_; while we expect *z*_e_~$V_{g}^{2/3}$ for large *V*_g_. This is the reason that the estimated resonance frequency from the previous modeling is much larger than the experimental results. In our model, we obtain *z*_e_ by performing several iterative calculations, to make the model accurate at a wide voltage range. Based on the iterative calculations, we solve the effective spring constant by considering both static deflection and the fundamental mode shape, which are largely unexplored in the previous model.

## 4. Conclusions

Using iterative computational modeling of device capacitance, we have developed comprehensive models for frequency tuning behaviors in doubly clamped and circular drumhead graphene resonators. We have also examined the effect of various parameters such as built-in tension, characteristic dimension, depth of air gap, number of layers, and device structure on frequency tuning, which provides useful guidelines for future design and fabrication to achieve new tunable graphene devices with the desired resonance frequencies and tuning ranges. 

## References

[1] Mak K.F., Sfeir M.Y., Wu Y., Lui C.H., Misewich J.A., Heinz T.F. (2008). Measurement of the optical conductivity of graphene. Phys. Rev. Lett..

[2] Wang L., Meric I., Huang P.Y., Gao Q., Gao Y., Tran H., Taniguchi T., Watanabe K., Campos L.M., Muller D.A. (2013). One-dimensional electrical contact to a two-dimensional material. Science.

[3] Lee C., Wei X., Kysar J.W., Hone J. (2008). Measurement of the elastic properties and intrinsic strength of monolayer graphene. Science.

[4] Lee G.-H., Cooper R.C., An S.J., Lee S., van der Zande A., Petrone N., Hammerberg A.G., Lee C., Crawford B., Oliver W. (2013). High-strength chemical-vapor–deposited graphene and grain boundaries. Science.

[5] Bunch J.S., van der Zande A.M., Verbridge S.S., Frank I.W., Tanenbaum D.M., Parpia J.M., Craighead H.G., McEuen P.L. (2007). Electromechanical resonators from graphene sheets. Science.

[6] Chen C.Y., Rosenblatt S., Bolotin K.I., Kalb W., Kim P., Kymissis I., Stormer H.L., Heinz T.F., Hone J. (2009). Performance of monolayer graphene nanomechanical resonators with electrical readout. Nat. Nanotechnol..

[7] Xu Y.H., Chen C.Y., Deshpande V.V., DiRenno F.A., Gondarenko A., Heinz D.B., Liu S., Kim P., Hone J. (2010). Radio frequency electrical transduction of graphene mechanical resonators. Appl. Phys. Lett..

[8] Singh V., Sengupta S., Solanki H.S., Dhall R., Allain A., Dhara S., Pant P., Deshmukh M.M. (2010). Probing thermal expansion of graphene and modal dispersion at low-temperature using graphene nanoelectromechanical systems resonators. Nanotechnology.

[9] Weber P., Güttinger J., Tsioutsios I., Chang D.E., Bachtold A. (2014). Coupling graphene mechanical resonators to superconducting microwave cavities. Nano Lett..

[10] Miao T., Yeom S., Wang P., Standley B., Bockrath M. (2014). Graphene nanoelectromechanical systems as stochastic-frequency oscillators. Nano Lett..

[11] Chen C., Lee S., Deshpande V.V., Lee G.-H., Lekas M., Shepard K., Hone J. (2013). Graphene mechanical oscillators with tunable frequency. Nat. Nanotechnol..

[12] Ye F., Lee J., Feng P.X.-L. (2018). Electrothermally tunable graphene resonators operating at very high temperature up to 1200 K. Nano Lett..

[13] Mathew J.P., Patel R.N., Borah A., Vijay R., Deshmukh M.M. (2016). Dynamical strong coupling and parametric amplification of mechanical modes of graphene drums. Nat. Nanotechnol..

[14] Alba R.D., Massel F., Storch I.R., Abhilash T.S., Hui A., McEuen P.L., Craighead H.G., Parpia J.M. (2016). Tunable phonon-cavity coupling in graphene membranes. Nat. Nanotechnol..

[15] Davidovikj D., Alijani F., Cartamil-Bueno S.J., van der Zant H.S.J., Amabili M., Steeneken P.G. (2017). Nonlinear dynamic characterization of two-dimensional materials. Nat. Commun..

[16] Güttinger J., Noury A., Weber P., Eriksson A.M., Lagoin C., Moser J., Eichler C., Wallraff A., Isacsson A., Bachtold A. (2017). Energy-dependent path of dissipation in nanomechanical resonators. Nat. Nanotechnol..

[17] Lee J., Krupcale M.J., Feng P.X.-L. (2016). Effects of γ-ray radiation on two-dimensional molybdenum disulfide (MoS_2_) nanomechanical resonators. Appl. Phys. Lett..

[18] He R., Feng X.L., Roukes M.L., Yang P. (2008). Self-transducing silicon nanowire electromechanical systems at room temperature. Nano Lett..

[19] Lee J., Wang Z., He K., Yang R., Shan J., Feng P.X.-L. (2018). Electrically tunable single- and few-layer MoS_2_ nanoelectromechanical systems with broad dynamic range. Sci. Adv..

[20] Xu Y.H., Li O.P., Xu R.M. Graphene resonant channel transistor. Proceedings of the IEEE International Wireless Symposium (IWS).

[21] Lekas M., Lee S., Cha W., Hone J., Shepard K. (2015). Noise modeling of graphene resonant channel transistors. IEEE Trans. Electron Devices.

[22] Mei T.D., Xu Y.H., Li O.P., Lan Y., Wu Y.Q., Xu R.M., Chen Y.F., Li Y.R. Accurate multi-bias equivalent circuit model for graphene resonant channel transistors. Proceedings of the IEEE International Microwave Symposium (IMS).

[23] Mei T.D., Xu Y.H., Lan Y., Li O.P., Sander M.R., Xu R.M., Li Y.R. (2017). A high-frequency compact model for graphene resonant channel transistors including mechanical nonlinear effects. IEEE Trans. Microw. Theory Tech..

[24] Chen C.Y. (2013). Graphene nanoElectroMechanical Resonators and Oscillators. Ph.D. Thesis.

[25] Schomburg W.K. (2011). Introduction to Microsystem Design.

